# Translation, Validity and Internal Consistency of the Quality of Dying and Death Questionnaire for Brazilian families of patients that died from cancer: a cross-sectional and methodological study

**DOI:** 10.1590/1516-3180.2022.0085.R2.09082022

**Published:** 2022-11-21

**Authors:** Bianca Sakamoto Ribeiro Paiva, Talita Caroline de Oliveira Valentino, Mirella Mingardi, Marco Antonio de Oliveira, Julia Onishi Franco, Michelle Couto Salerno, Helena Palocci, Tais Cruz de Melo, Carlos Eduardo Paiva

**Affiliations:** I PhD. Researcher and Professor, Palliative Care and Quality of Life Research Group (GPQual), Postgraduate Program, Hospital de Câncer de Barretos, Barretos (SP), Brazil.; II MSc. Nurse and Doctoral Student, Palliative Care and Quality of Life Research Group (GPQual), Postgraduate Program, Hospital de Câncer de Barretos, Barretos (SP), Brazil.; III RN. Nurse and Master's Student, Palliative Care and Quality of Life Research Group (GPQual), Postgraduate Program, Hospital de Câncer de Barretos, Barretos (SP), Brazil.; IV MSc. Biostatistics, Palliative Care and Quality of Life Research Group (GPQual), Postgraduate Program, Hospital de Câncer de Barretos, Barretos (SP), Brazil.; V MD. Physician, Dr. Paulo Prata, School of Health Sciences of Barretos and Palliative Care and Quality of Life Research Group (GPQual), Postgraduate Program, Hospital de Câncer de Barretos, Barretos (SP), Brazil.; VI RN. Research Nurse, Palliative Care and Quality of Life Research Group (GPQual), Postgraduate Program, Hospital de Câncer de Barretos, Barretos (SP), Brazil.; VII MD. Physician, Dr. Paulo Prata, School of Health Sciences of Barretos and Palliative Care and Quality of Life Research Group (GPQual), Postgraduate Program, Hospital de Câncer de Barretos, Barretos (SP), Brazil.; VIII MD. Physician, Dr. Paulo Prata, School of Health Sciences of Barretos and Palliative Care and Quality of Life Research Group (GPQual), Postgraduate Program, Hospital de Câncer de Barretos, Barretos (SP), Brazil.; IX PhD. Physician and Professor, Palliative Care and Quality of Life Research Group (GPQual), Postgraduate Program, Hospital de Câncer de Barretos, Barretos (SP), Brazil.

**Keywords:** Validation study, Neoplasms, Death, The quality of dying and death, Cancer, Questionnaire

## Abstract

**BACKGROUND:**

The Quality of Dying and Death Questionnaire (QoDD) may prove to be an important evaluation tool in the Brazilian context, and, therefore, can contribute to a more precise evaluation of the dying and death process, improving and guiding the end-of-life patient care.

**OBJECTIVE:**

To translate and cross-culturally adapt the QoDD into Brazilian Portuguese and measure its validity (convergent and known-groups) and internal consistency

**DESIGN AND SETTING:**

A cross-sectional, methodological study was conducted at the Hospital de Câncer de Barretos, Brazil

**METHODS:**

A total of 78 family caregivers participated in this study. Semantic, cultural, and conceptual equivalences were evaluated using the content validity index. The construct validity was assessed through convergent validation and known groups analysis [presence of family members at the place of death; feel at peace with dying; and place of death (hospital versus home; hospital versus Palliative Care)]. Internal consistency was evaluated using Cronbach's alpha.

**RESULTS:**

The questionnaire was translated into Brazilian Portuguese and presented evidence of a clear understanding of its content. Cronbach's alpha values were ≥ 0.70, except for the domains of treatment preference (α = 0.686) and general concerns (α = 0.599). The convergent validity confirmed a part of the previously hypothesized correlations between the Palliative Care Outcome Scale-Brazil (POS-Br) total scores and the QoDD domain scores. The QoDD-Br domains could distinguish the patients who died in palliative care and general wards.

**CONCLUSION:**

The QoDD-Br is a culturally adapted valid instrument, and may be used to assess the quality of death of cancer patients.

## INTRODUCTION

The death process is subjectively determined and may be influenced by cultural factors, individual judgments, type and stage of the underlying disease, and the social and professional role with respect to the death experience.^1^ The interest in promoting a “good death” has been increasingly discussed, mainly due to the increase in life expectancy of the population and advances in medicine.^1^ The Institute of Medicine Committee on Care at the End of Life characterized high-quality death as “death free from avoidable anguish and suffering for patients, families and their caregivers, according to the wishes of patients and caregivers and in line with clinical, cultural and ethical standards.”^2,3^ The end-of-life stage leads to changes, which allows the development of standards that improve the quality of death (QOD). Simultaneously, a “good death” is equivalent to a death consistent with the patient's personality^4^ and autonomy.

Therefore, QOD may be defined as the assessment of the last days of life and the moment of death, respecting the way that moment is prepared, faced, experienced and dealt with by those who have known terminal illness.^5^ Different authors provide varied criteria for determining the QOD, such as reaffirming the need to prioritize the absence of pain during the end-of-life period. However, there is a consensus that the quality of death and dying is greater than the control of physical symptoms (such as pain), since there are multiple dimensions inherent to this process.^6–9^ Therefore, practical measures are necessary to improve this indicator in the Brazilian context.

Among all the instruments of the QOD assessment described in the literature, the “Quality of Dying and Death Questionnaire” (QoDD) is the most widely studied and best validated.^1,10,11^ It was developed by Patrick et al.^4^ due to a shortage of instruments for assessing the QOD. The study expected to provide a better evaluation of post death reports and the experience regarding the QOD and dying, as well as to evaluate the interventions that improve the quality of care at the end of life.

The QoDD has been adapted in different languages and cultures and has demonstrated greater validity and reliability than other questionnaires.^11,12^ It presents satisfactory psychometric results, with an internal consistency of 0.88 and test-retest reliability of 0.7 in studies conducted in Germany and Spain.^3,13^ However, no Portuguese version has been culturally adapted and validated in the Brazilian population. The QoDD may prove to be an important evaluation tool in the Brazilian context, and thus, may contribute to a more precise evaluation of the dying and death process, improving and guiding the end-of-life patient care.

## OBJECTIVE

The purpose of this study was to translate and cross-culturally adapt the QoDD into Brazilian Portuguese and measure its validity (convergent and known-groups) and internal consistency.

## METHODS

### Study design

This was a descriptive, cross-sectional, and methodological study.

### Setting

The study was conducted at Hospital do Câncer de Barretos (Barretos, São Paulo, Brazil), a reference hospital in Latin America for cancer treatment. It is an assistential, teaching, and research institution.

### Patient and public involvement statement

Caregivers (family members) were not involved in the design or planning of the study; however, were informed regarding the nature and purpose of this study. Authorization for participation was obtained in the form of signed consent forms from the primary family caregiver. The entire validation process was performed following the permission of one of the authors of the original QoDD.^10^

### Phase I - Translation and cultural adaptation process

The cross-cultural adaptation of the QoDD was initiated after obtaining permission from the author of the original version.^10^ International methodology adopted for the translation and cultural adaptation included translation, a synthesis of the translations, back-translation, an expert panel, and a pretest according to the methodology proposed by Beaton et al.^14^ and Souza and Rojjanasrirat.^15^

Initially, the original questionnaire was translated from English into Portuguese by two independent translators, both native English speakers, without the knowledge of the issues addressed by the QoDD. The translated versions of the questionnaire were coded as T1 and T2.

The second step included a synthesis meeting of four specialized professionals: a doctor experienced in palliative care (PC), a researcher experienced in the QOD, and two other professionals in the research field experienced in the care practice. In this step, a synthesized version (T12) was generated from the evaluation of T1 and T2 translations. Each aspect of the translations was analyzed and discussed to achieve a consensus between the two versions, ensuring equivalence.

Next, the instrument was back translated from Portuguese into the original language. Two independent translators performed the back-translations (BT1 and BT2), one American with fluency in Portuguese and the other, a native Brazilian with expertise in the English language.

An expert committee meeting was conducted during which all the material produced in the previous steps was analyzed. This team of five experts included a clinical oncologist, a palliative physician, a research nurse in PC, a research psychologist in PC and a biostatistician experienced in the validation of assessment instruments. The committee's main objective was to produce a final version of the tool that would be culturally adapted for use during the pretesting. To assess the representativeness of each item, a Likert scale with scores between 1 and 4 was used. The content validity index (CVI) was calculated by summing the equivalences of the items and dividing it by the total number of items. A minimum value of 0.80 was accepted for the evaluated item to be considered appropriate.^15^

The pre-testing phase included 26 family caregivers who were >18 years, of either sex, considered the primary caregiver, aware that the patient's death was from cancer, and knew how to read. Family caregivers with significant hearing loss that prevented them from telephonic communication were excluded. The family caregivers were contacted via telephone within 4-12 weeks after the date of death of their loved one.

**Figure 1:**
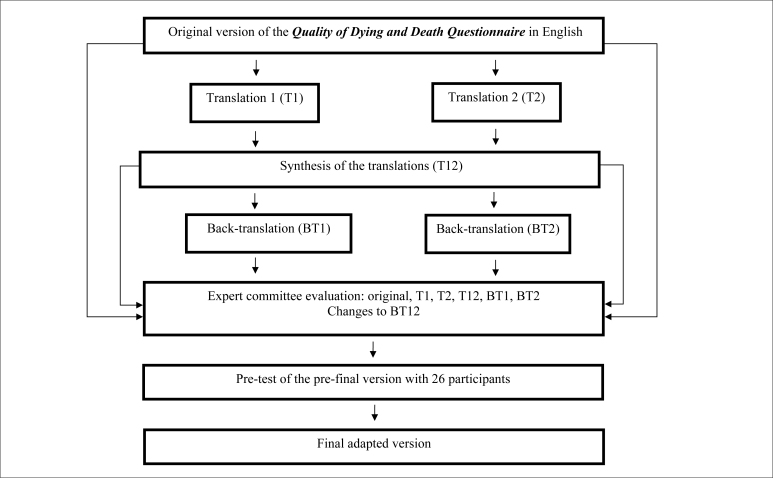
Flowchart of the translation and cross-cultural adaptation steps.

Figure 1 shows the steps of the translation and cross-cultural adaptation process.

### Phase II - Assessment of psychometric properties

A different sample of family caregivers was contacted by phone to measure the psychometric properties of the QoDD-Br. One of the measures of reliability - internal consistency - was assessed using Cronbach's alpha. Values from 0.70-0.95 were considered to be adequate.^16^ For convergent construct validity, correlations between the QoDD-Br and the Palliative Care Outcome Scale-Brazil (POS-Br; an assessment tool designed to address multidimensional aspects of palliative care, such as physical and psychological symptoms, spiritual considerations, practical concerns, and emotional and psychosocial needs)^17,18^ scores were hypothesized a priori by the researcher's judgment based on a clinical routine and the literature. Correlations with values ≥ 0.4 (moderate to highly strong) were considered acceptable.^19,20^ In the known-groups analysis, the groups were compared using the mean (standard deviation) of each domain, as measured by the QoDD-Br, to assess whether the instrument could discriminate between the groups as hypothesized.

Primary family caregivers of patients who died from cancer and were > 18 years of age were invited to participate in the study's validation step. They were selected through telephone contact, and they consented to answer the QoDD-Br questionnaire adapted to the Brazilian culture and the POS-Br. To preserve their mental health and avoid the worsening of their psychological condition due to participation in the study, the Patient Health Questionnaire-9 (PHQ-9)^21^ was administered to screen for depressive symptoms and suicide risk. Family caregivers who selected option 1 - several days, 2 - more than half the days or 3 - almost every day in question 9 of the PHQ-9 questionnaire (suicidal ideation) or had a total score ≥ 12, were excluded.^22^

### Instruments

#### Quality of Dying and Death Questionnaire (QoDD)

It comprised 31 items divided into six domains measuring aspects related to symptoms and personal control, preparation for death, family concerns, treatment preferences, whole person concerns, and moment of death. It takes into account the experiences in the last seven days of the patient's life and the state of the patient. The response scale used is a Likert-type scale with scores varying from 0 to 100, where higher scores indicate better QOD.^10^

#### Palliative Care Outcome Scale - Brazil (POS-Br)

The POS-Br is a tool largely used to measure the quality of life (QOL) during the last 3 days of the patient's life,^20^ as well as of patients who undergo PC. This scale consists of 11 items. The answers are provided on a five-point Likert scale, except item 9, which has three points, and an open question regarding the main problems experienced by the patient. The POS-Br scores range from 0 to 40 points, with 0 representing the best QOL and 40 representing the worst QOL.^17,18^

#### Patient Health Questionnaire-9 (PHQ-9)

The PHQ-9 is a useful tool for the screening of depressive symptoms. It includes nine questions, rated on a four-point Likert scale (0 to 3), for a possible total of 27 points. The final score classification is as follows: 0–4, without depression; 5–9, mild depression; 10–14, moderate depression; 15–19, moderate to severe depression; and > 20, severe depression.^21^

#### Calculation of sample size

Sample calculation for the pretest phase followed the methodology described by Beaton et al., which advocates the participation of 10–40 participants.^14^ In this study's pre-testing phase, 33 individuals participated. For construct validity, the sample size calculation was a minimum of 50 patients.^16^

#### Statistical analyses

Internal consistency was assessed using Cronbach's alpha coefficient, and a value between 0.70 and 0.95 was considered adequate.^16^ Convergent validity correlations were measured using Pearson's correlation coefficient (r > 0.4).^19^ The calculation was performed considering total scores on the QoDD-Br and POS-Br instruments and subsequently the scores in their domains. For the known-groups analysis, the groups were compared using the nonparametric Mann–Whitney U test. It was expected that the QoDD-Br would discriminate between the caregiving groups in accordance with the hypotheses based on the following factors: presence of family members at the place of death (yes versus no); feel at peace with dying (yes versus no); and place of death (hospital versus home; hospital versus PC hospital).

The data were stored on the REDCap Plataform^23^ and evaluated using the IBM-SPSS software, version 21.0 (IBM Corp., Armonk, New York, United States). The significance level was set at 0.05.

### Ethical aspects

The study was approved by the Committee of Ethics in Research of Hospital de Câncer de Barretos, under opinion n. 1329/2017 (May 18, 2017). All the participants invited to participate in the study signed the free and informed consent form.

## RESULTS

### Phase I - Translation and cultural adaptation process translation

The title and 31 items were translated with similar meaning, with no grammatical or semantic distinctions between T1 and T2. The original acronym was maintained, and Br was added to identify the Brazilian instrument: QoDD-Br. The back-translations (BT1 and BT2) did not indicate significant conceptual changes or inconsistencies in the translation process and were useful in guiding effective and consistent actions in the expert committee step.

Table 1 shows the items questioned by the expert committee and their respective equivalences. From the 112 questionnaire items evaluated, there was a 100% agreement on 104 items classified with scores of 3 and 4; CVI = 1. Items 1, 2, 18, 19, 34, 42, 78 and 99 received a score of 2, and thus, changes suggested by the experts were necessary.

**Table 1:** Description of the items with modifications requested by the expert committee

Items	Statement or question	Equivalences			Total CVI
		Semantic	Cultural	Conceptual	
**1**	Quality of Dying and Death (QODD) Questionnaire	0.8	1	1	0.93
**2**	QoDD: Interview following the death of a loved one	0.6	0.6	0.8	0.67
**18**	7 recall days 7	0.8	1	0.8	0.87
**19**	30 recall days 30	0.8	1	0.8	0.87
**34**	Each question has two parts. The first part will ask you how often X experienced each item using a scale where 0 is “none of the time” and 5 is “all of the time”	0.6	1	1	0.87
**37**	Let us start with an example. In the last month of her/his life, how often did X listen to music? I would like you to use the first scale to tell me how often X listened to music during the last month of her/his life, with 0 being “none of the time” and 5 being “most of the time”	0.8	1	1	0.93
**42**	How often did X appear to have control over what was going on around her/him?	0.8	1	1	0.93
**78**	Where did your loved one die?	1	1	0.8	0.93
**99**	What is the highest school level you completed?	1	0.8	1	0.93

Equivalences calculated by the content validity index (CVI).

Each interview was conducted over telephone and lasted approximately 30 minutes. The initial sample included 33 participants; two refused to participate due to lack of time, and five were excluded because they reported that they were not prepared to remember the death of their loved ones. The sociodemographic characteristics of the family caregivers and patients are shown in Table 2. All the participants answered a questionnaire with questions related to the understanding of each item.

**Table 2:** Sociodemographic characteristics of the participants who completed the pretest

Variable		Family caregivers		Patients	
		n	%	n	%
**Sex**	Female	22	84.6	14	53.8
	Male	4	15.4	12	46.2
**Race**	White	16	61.5	20	77.0
	Black	3	11.5	1	3.8
	Mixed	7	27.0	5	19.2
**Education**	Primary	6	23.0	7	27.0
	Secondary	12	46.0	14	54.0
	Higher	8	31.0	5	19.0
**Religion**	Catholic	19	73.1	19	73.1
	Evangelical	5	19.2	5	19.2
	Spiritist	2	7.7	2	7.7
**Civil Status**	Married	14	53.8	15	57.7
	Single	7	27.0	4	15.4
	Widowed	3	11.5	6	23.1
	Separated	2	7.7	1	3.8
**Place of death**	Acute Care Hospital	-	-	1	3.8
	PC Hospital^1^	-	-	25	96.2
**Relatives**	Father	1	3.8		
	Mother	1	3.8	-	-
	Son/Daughter	9	35.0	-	-
	Nephew/Niece	2	7.7	-	-
	Son/Daughter -in-law	2	7.7		
	Husband/Wife	6	23.0	-	-
	Others^2^	5	19.0	-	-
**Time interval between patient's death and pretest (days)**	Mean (SD)	76.12 (27.71)			

SD = standard deviation.

1 PC Hospital: hospital exclusively dedicated to patients in palliative care;

2 Others: Friend, stepdaughter, boyfriend or cousin.

Annex 1 shows the final version of the QoDD-Br to Brazilian culture.

### Phase II - Assessment of psychometric properties

A total of 566 family caregivers were screened as potential participants. Of these, only 114 answered the telephone calls during which they were invited to participate in the study. Twelve (10.5%) family caregivers refused to participate in the study and 50 (43.8%) were excluded due to depression symptoms identified by the PHQ-9. Thus, final sample included 52 (45.6%) family caregivers

Among the included family caregivers (n = 50), 41 (78.8%) were women, 25 (48.1%) were married, 18 (34.6%) were children, and 17 (32.7%) were spouses. Regarding the characteristics of the patients, 37 (32.5%) had gastrointestinal cancer, 19 (16.7%) had lung cancer, 10 (8.8%) had head and neck cancer, and 10 (8.8%) had hematological cancer. Seven (13.5%) of the patients died at home.

Regarding internal consistency, most QoDD-Br domains presented Cronbach's α values ≥ 0.70, with the exception of the treatment preference (α = 0.686) and general concerns (α = 0.599) domains (Table 3). Regarding convergent validity, the a priori hypothesized correlations were confirmed between the domains “preparation for death” (r = −0.422, P = 0.002), “symptoms and personal control” (r = −0.465, P = 0.001), and “whole person concerns” (r = −0.405, P = 0.003). The correlations between the QoDD-Br and POS-Br scores are summarized in Table 4. Researchers expected that the instrument could discriminate the presence of family members at the place of death (yes versus no), feeling at peace with dying (yes versus no), and place of death (hospital versus home; hospital versus PC hospital). The known-groups analysis showed that the instrument could discriminate between the family caregiver groups, as shown in Table 5.

**Table 3:** Internal consistency of the Quality of Dying and Death Questionnaire-Brazilian (QoDD-Br)

QoDD-Br domains	Cronbach's alpha
Symptoms and personal control	0.825
Preparation for death	0.776
Moment of death	0.814
Family	0.742
Treatment preference	0.686
Overall person concerns	0.599
**QoDD-Br total score**	0.955

**Table 4:** Convergent validation (correlations) between the Quality of Dying and Death Questionnaire-Brazilian (QoDD-Br) domains and Palliative Care Outcome Scale-Brazil (POS-Br)

QoDD-Br domains	POS-Br total	P value
Symptoms and personal control	**-0.465**	0.001
Preparation for death	**-0.422**	0.002
Moment of death	-0.358	0.009
Family	-0.125	0.377
Treatment preference	-0.045	0.754
Overall person concerns	**-0.405**	0.003
**QoDD-Br total score**	-0.242	0.290

Correlation with a coefficient above 0.4.

**Table 5:** Mean comparison of domains measured by Quality of Dying and Death Questionnaire-Brazilian (QoDD-Br) version between family caregivers' groups (known-groups analysis)

Variables	Category	QoDD-Br Domains												QoDD-Br Total score	
		Symptoms and personal control		Preparation for death		Moment of Death		Family		Treatment preference		Overall person concerns			
		Mean (SD)	P	Mean (SD)	P	Mean (SD)	P	Mean (SD)	P	Mean (SD)	P	Mean (SD)	P	Mean (SD)	P
Presence of family members at the place of death	No	38.57 (13.28)	0.253*	54.68 (17.02)	0.124*	34.76 (30.78)	*0.023*	65.69 (28.06)	0.329	70.24 (25.65)	0.557	58.10 (17.39)	0.098	53.47 (16.10)	0.065*
	Yes	50.72 (27.11)		67.09 (19.81)		71.37 (32.41)		76.36 (23.77)		75.68 (26.68)		70.26 (22.44)		66.77 (17.42)	
Feel at peace with dying	No	41.79 (24.74)	0.180*	56.75 (17.38)	*0.036* *	48.33 (34.11)	*0.017*	72.30 (24.15)	0.474	74.05 (28.01)	0.724	58.04 (19.84)	0.005	56.31 (18.39)	*0.013* *
	Yes	52.99 (26.39)		69.87 (19.81)		72.35 (33.36)		75.99 (24.28)		78.64 (21.64)		75.20 (20.92)		70.00 (15.66)	
Place of death	Hospital	50.65 (26.11)	0.387	64.83 (20.16)	0.589*	62.63 (35.17)	0.072	74.11 (25.10)	0.678	73.90 (27.64)	0.762	73.93 (13.30)	0.500*	64.40 (18.56)	0.652*
	Home	39.05 (24,03)		69.23 (17.97)		90.95 (11.01)		80.14 (19.72)		81.43 (15.97)		67.80 (23.15)		67.70 (11.96)	
Place of death	PC Hospital	55.16 (27.41)	0.178*	72.83 (19.01)	*0.001*	71.03 (33.24)	0.052	80.16 (22.78)	*0.044*	82.56 (21.93)	*0.017*	75.10 (20.74)	*0.012* *	70.32 (17.96)	*0.015* *
	Hospital	44.47 (23.52)		53.87 (16.49)		51.14 (35.34)		65.84 (26.35)		61.39 (30.74)		57.81 (23.03)		56.50 (16.71)	

SD = standard deviation. Statistical analyses were performed using nonparametric Mann–Whitney U test.

* U-test. Statistically significant P values at the 0.05 level are in italics.

## DISCUSSION

This study translated, culturally adapted, and validated the QoDD for use in the Brazilian population.^14,24^ The QoDD may prove to be an important evaluation tool in Brazil, contributing to a more accurate assessment of the death and dying process and improving the quality of life and death of cancer patients at the end of life.

Several tools have been developed in an attempt to quantify/characterize QOD, including the Good-Death Scale, the Good Death Inventory (GDI), the Quality of Dying in Long-term Care (QOD-LTC), the Client Generated Index tool (CGI), the McGill Quality of Life questionnaire (MQOL) and the QoDD. The QoDD is the most widely used tool and has demonstrated greater validity and reliability than other instruments.^11^

The QODD has been widely used in QOD assessment, used and validated in different health care settings, such as in palliative care and Intensive Care Units. It is used to assess the QOD of patients reported by their family caregivers based on the six important domains of QOD symptoms: personal control, preparation for death, family concerns, treatment preferences, whole person concerns, and moment of death^4,25^

Each society has its own behaviors, beliefs, attitudes, customs and social habits that must be considered in a translation and cross-cultural adaptation process.^15,26^ During this process, it is possible to identify possible translation failures, that if left unresolved, may result in difficulties in the utilization of the construct and conduction of intercultural comparative studies.^14^

As with the previous studies^24,27,28^ internal consistency was also considered satisfactory (α = 0.95). In contrast, the Cronbach's α coefficients for the domains “treatment preference” and “whole person concerns” were both below 0.7 (α = 0.686 and α = 0.599, respectively). However, the comparison with the previous studies is limited, as the other studies did not report the Cronbach's α values for the QoDD domains.

Two previous validations conducted correlation analyses between the QoDD and POS scores. Both studies found negative correlation coefficients (r > 0.4) between the total QoDD and POS scores. Although a significant correlation between the two measures was not observed, the following three QoDD domains had significant correlations with the POS total score: “symptoms and personal control;” “preparation for death;” and “whole person concerns.” Unfortunately, comparisons of the POS correlations with the QoDD domains have not been previously reported, which makes comparisons difficult. In considering the QoDD a multidimensional tool, it was believed that the results should be presented not only for the total score, but also mainly for its domain scores.^24,27^

In the known-groups analysis, the QoDD was able to discriminate distinct groups of patients as hypothesized. It should be noted that the QOD scores were higher in patients cared for by palliative care specialized teams than in patients who died in wards not specialized in PC. In contrast, unlike this study's hypothesis, there was no difference in scores between dying in the hospital or at home. This may be explained by the fact that patients who died at home were not cared for by a home care team. Many Brazilian patients face socio-economic difficulties (for e.g., poverty or lack of food and medicine) that can limit their end-of-life care conditions in addition to the poor access to palliative care, which should be offered by primary care teams.

The QoDD does not make it possible to assess the death and dying wishes of the patients, so it depends on the family caregivers. This evaluation is related to the memories of family caregivers in retrospective evaluation reports, but memory, emotions, and other person-related factors may bias their reports.^29,30^ To minimize these effects, the family caregivers were contacted at least 4 weeks and no later than 12 weeks after the death of the patient.

The strength of this study is the QoDD application method, which was performed through telephonic communication. This type of contact allows the caregivers to be interviewed without needing to leave their residence to participate in the interview. Since Brazil is a continental country and considering that most family members return to their cities of origin after the patient's death, a QOD questionnaire valid for usage via telephone is certainly of great clinical utility.

Taking into account that Brazil is still a country with a poor QOD,^31^ it is urgent to adequately measure the QOD so that measures may be adopted at the local and public health levels. The QoDD-Br could be used as an indicator of the quality of care and to compare different health care services. It may be an useful tool to measure improvements after interventions such as staff training, after the change in protocols and availability of financial resources.

This study has a few limitations. It was restricted to only one center in Brazil in a city located in the interior of São Paulo state. However, despite the great geographic expansion of the country, all five regions share the same language, and although there are certain cultural variations, this is not a factor that hampers the generalization power of the instrument to the Brazilian population as a whole. Other psychometric properties were not evaluated, including construct validity, reliability (intra- and inter-rater reliability), and measurement error. Although a wide variety of psychometric properties may be assessed, they are not necessarily investigated in all validation studies. Thus, different validation studies may even be complementary for evaluating the same instrument.

## CONCLUSION

The QoDD-Br was culturally adapted and the psychometric properties of the convergent and known-groups validities, as well as the internal consistency were analyzed. In general, the items were adequately understood by the caregivers, and the psychometric properties were considered adequate. The QoDD-Br is ready to be used as a new indicator of the quality of the dying process in Brazil. Further studies with larger sample sizes should be conducted to provide a confirmatory factor analysis, others measures of reliability, standard error of measurement, minimal detectable change, and responsiveness analysis.

## Annex 1 Original QoDD and version adapted to the Brazilian culture

**Table:**

Item	QoDD original version	QoDD-Br
1	*How often did X appear to have her/his pain under control?*	Com que frequência X parecia ter a dor dela/dele sob controle?…
2	*How often did X appear to have control over what was going on around her/him?*	Com que frequência X parecia ter consciência do que está acontecendo ao redor dela / dele?
3	*How often was X able to feed herself/himself?*	Com que frequência X foi capaz de alimentar-se?
4	*How often did X have control of her/his bladder or bowels?*	Com que frequência X teve controle da bexiga ou do intestino?
5	*How often did X breathe comfortably?*	Com que frequência X respirava confortavelmente?
6	*How often did X appear to feel at peace with dying?*	Com que frequência X parecia sentir-se em paz com o fato de morrer?
7	*How often did X appear to be unafraid of dying?*	Com que frequência X parecia não ter medo de morrer?
8	*How often did X laugh and smile?*	Com que frequência X ria e sorria?
9	*How often did X appear to have the energy to do most things that s/he wanted to do?*	Com que frequência X parecia ter energia para fazer a maioria das coisas que ela / ele queria fazer?
10	*How often did X appear to be worried about strain on her/his loved ones?*	Com que frequência X parecia estar preocupado (a) sobre o que seus entes queridos sentem?
11	*How often did X appear to keep her/his dignity and self-respect?*	Com que frequência X parecia manter sua dignidade e autorrespeito?
12	*How often did X spend time with her/his spouse or partner?*	Com que frequência X passava tempo com seu cônjuge ou parceira (o)?
13	*How often did X spend time with her/his children?*	Com que frequência X passava seu tempo com seu (s) filhos (as)?
14	*How often did X spend time with other family and friends?*	Com que frequência X passava seu tempo com outros familiares e amigos?
15	*How often did X spend time alone?*	Com que frequência X passava seu tempo sozinha (o)?
16	*How often did X spend time with pets?*	Com que frequência X passava seu tempo com animais de estimação
17	*Did X appear to find meaning and purpose in her/his life?*	X parecia ter encontrado sentido e propósito na vida dela/dele?
18	*Was X touched or hugged by her/his loved ones?*	X foi tocada (o) ou abraçada (o) pelos entes queridos dela/dele?
19	*Did X attend any important events - for example, weddings, graduations, and birthdays?*	X participou de algum evento importante – por exemplo, casamentos, formaturas e aniversários?
20	*Were all of X's health care costs taken care of?*	Todos os custos dos cuidados de saúde de X foram resolvidos?
21	*Did X say goodbye to the loved ones?*	X disse adeus aos seus entes queridos?
22	*Did X have one or more visits from a religious or spiritual advisor?*	X recebeu uma ou mais visitas de um conselheiro espiritual ou religioso?
23	*Did X have a spiritual service or ceremony before his/her death?*	X teve um serviço ou cerimônia espiritual antes de morrer?
24	*Was a mechanical ventilator or kidney dialysis used to prolong X's life?*	Ventilação mecânica ou hemodiálise foi usada para prolongar a vida de X?
25	*Did X have the means to end her/his life if s/he needed to?*	X tinha meios para dar um fim à vida dele (a) se ele (a) quisesse?
26	*Did X clear up any bad feelings with others?*	X esclareceu quaisquer sentimentos ruins com os outros?
27	*Did X have her/his funeral arrangements in order prior to death?*	X deixou serviço funerário dela/dele preparado antes de sua morte?
28	*Did X discuss her/his wishes for end-of-life care with her/his doctor – for example, resuscitation or intensive care?*	X discutiu seus desejos para os cuidados de fim de vida com o médico dele (a) - por exemplo, ressuscitação ou cuidado intensivo?
29	*Where did your loved one die?*	Onde seu ente querido morreu?
30	*Was anyone present at the moment of X's death?*	Alguém estava presente no momento da morte de X?
31	*In the moment before the death of X, s/he was …*	No momento antes da morte de X, ela/ele estava …
